# Tuberculosis Management by Private Practitioners in Mumbai, India: Has Anything Changed in Two Decades?

**DOI:** 10.1371/journal.pone.0012023

**Published:** 2010-08-09

**Authors:** Zarir F. Udwadia, Lancelot M. Pinto, Mukund W. Uplekar

**Affiliations:** 1 Department of Respiratory Diseases, P. D. Hinduja National Hospital and Medical Research Centre, Mumbai, India; 2 Stop TB Department, World Health Organization, Geneva, Switzerland; McGill University, Canada

## Abstract

**Setting:**

Mumbai, India. A study conducted in Mumbai two decades ago revealed the extent of inappropriate tuberculosis (TB) management practices of private practitioners. Over the years, India's national TB programme has made significant progress in TB control. Efforts to engage private practitioners have also been made with several successful documented public-private mix initiatives in place.

**Objective:**

To study prescribing practices of private practitioners in the treatment of tuberculosis, two decades after a similar study conducted in the same geographical area revealed dismal results.

**Methods:**

Survey questionnaire administered to practicing general practitioners attending a continuing medical education programme.

**Results:**

The participating practitioners had never been approached or oriented by the local TB programme. Only 6 of the 106 respondents wrote a prescription with a correct drug regimen. 106 doctors prescribed 63 different drug regimens. There was tendency to over treat with more drugs for longer durations. Only 3 of the 106 respondents could write an appropriate prescription for treatment of multidrug-resistant TB.

**Conclusions:**

With a vast majority of private practitioners unable to provide a correct prescription for treating TB and not approached by the national TB programme, little seems to have changed over the years. Strategies to control TB through public sector health services will have little impact if inappropriate management of TB patients in private clinics continues unabated. Large scale implementation of public-private mix approaches should be a top priority for the programme. Ignoring the private sector could worsen the epidemic of multidrug-resistant and extensively drug-resistant forms of TB.

## Introduction

Two decades ago, a study assessing TB management practices of private medical doctors was conducted at Dharavi, one of Asia's most densely populated slums located in Mumbai, India[1]. Revealing that 100 practitioners had prescribed 80 different drug regimens for treatment of TB, the study highlighted the magnitude of the poor prescribing practices of private medical practitioners. The study also seemed to help stimulate national and global efforts to educate and engage such practitioners in TB control[2], [3], [4].

India's Revised National Tuberculosis Control Programme (RNTCP) has made significant progress in TB control over the last decade through countrywide DOTS implementation[5], [6].This also includes efforts to engage the private medical sector in TB care and control through various published schemes[2].Two factors aroused our interest in exploring the change in prescribing practices by private practitioners over the past two decades since the original study was published. First, we found an alarming trend of increasing incidence of multidrug-resistant (MDR-TB) among samples sent to the laboratory at the P D Hinduja Hospital, a modern medical centre in Mumbai[7]. The incidence of MDR-TB among treated cases was 60 percent[8]. Secondly, this cohort of patients had received a significant number of irrational prescriptions prior to consulting our hospital. Clearly, the need for another audit of private practitioners prescriptions for TB seemed as pertinent as it was two decades ago. In order to understand any changes in the TB management practices of private practitioners, we conducted our study on private practitioners practicing in the same geographical area.

## Materials and Methods

The study was carried out among private practitioners practicing at Dharavi, a large slum in Mumbai. Dharavi covers an area of approximately 1.75 sq. kilometers, and has a population of over 2.5 million. A list of all private medical practitioners practicing in the area was compiled from those available with the local medical association and representatives of drug companies. The practitioners belonged to and practiced different systems of medicine. The Hinduja hospital regularly offers Continuing Medical Education(CME) sessions to practicing physicians. The listed practitioners were invited for a CME session on management of tuberculosis at a venue close to the slum area. Participation was voluntary and no incentives were offered for participation. Prior to the beginning of the CME session, a short questionnaire was handed out to each of the attending practitioners. Besides seeking general information related to their private practice, a main question posed to private practitioners was identical to the one asked in the original study^1^ (*“Please write a prescription for a previously untreated adult case of sputum-positive pulmonary tuberculosis weighing 50 kg”*). In addition, a question was also posed to understand their prescribing practices in the treatment of MDR TB (*“Please write a prescription for a previously untreated adult case of multidrug-resistant tuberculosis resistant to isoniazid and rifampicin and weighing 50 kg”*). The practitioners were expected to write a prescription and specify drugs, dosage and duration of treatment in three columns provided. The prescriptions written by the practitioners were then analyzed and compared for appropriateness with those recommended nationally and internationally[9], [10].The prescribing practices were also compared with those reported in the study published in 1991[1].

## Results

Of the 150 physicians listed and invited for the CME, 106 attended it and all of them consented to participate in the study. 46 practitioners were qualified to practice western medicine and are referred to here as allopaths while 60 were trained in one of the alternative systems of medicine—homeopathy, ayurveda or unani—and are referred to here as non-allopaths. Their training background regardless, 26 (44%) of the non-allopathic physicians admitted that they practiced mainly Western medicine in their clinics. Most participating physicians had over five years in clinical practice and the median number of TB patients seen per month by a practitioner was eight. Only 20 of the 104 respondents claimed to be using drugs from alternative systems of medicine in the treatment of TB in addition to the modern drugs. A third of responding practitioners said they referred their TB patients to government services for treatment. Table 1 gives a summary of other details of the participating private practitioners.

**Table 1:** Demographics and practice details.

		Number of physicians
**Number of years in practice**	≤5	17
	6–15	35
	16–25	17
	≥25	30
	No response	7
		
**Number of new TB patients seen every month?**		
	<1	9
	1–5	64
	6–10	20
	11–20	3
	>20	3
	No response	7
**Use drugs of alternative systems of medicine to treat TB?**	Yes	20
	No	84
	No response	2
**Information source to update knowledge on TB management**	Books	25
	Internet	19
	CMEs	43
	Journals	28
	Pharmaceutical representatives	25

In response to the question on the prescription they would write for a new adult patient with drug-susceptible TB, only 6 of the 106 respondents prescribed what could be considered an appropriate drug regimen with correct drugs, dosage and duration. Overall, 106 doctors prescribed 63 different drug regimens for treatment of TB. No practitioner prescribed intermittent drug regimen currently recommended by the RNTCP. The various regimens prescribed by the physicians are summarized in Table 2. There were no significant differences between the prescriptions offered by doctors trained in Western medicine and those trained in alternative systems (Table 3). However, significant differences were observed when the prescriptions for the treatment of drug susceptible TB were compared with those reported in the 1991 study. If only those regimens with four anti-TB drugs in the intensive phase and a total duration of minimum six and maximum eight months duration were considered appropriate, a significantly higher proportion of prescriptions in the current study were appropriate compared to the 1991 study (47% against 13%, p-value<0.001). Over a half of all prescriptions in this study were still inappropriate however.

**Table 2:** Drug regimens prescribed by private practitioners.*

HRE	6	2
HREZ	2	4
HREZ	3	3
HREZ	5	1
HREZ	6	34
HREZ	7	3
HREZ	8	10
HREZ	9	21
HREZ	10	1
HREZ	12	3
HREZ	not mentioned	2
SHRE	18	1
SHREZ	6	2
SHREZ	8	2
SHREZ	9	5
SHREZ	12	1
SHREZ	not mentioned	2
HREZ+Levofloxacin	8	3
HREZ+Levofloxacin	9	1
No response	—	5

*Abbreviations used for drugs prescribed: S- Streptomycin, H- Isoniazid, R- Rifampicin, E – Ethambutol, Z – Pyrazinamide.

**Table 3:** Comparison between prescriptions of allopaths and physicians trained in alternative forms of medicine.

*PARAMETER COMPARED*	Allopath	Non-allopath	Not mentioned
**Correct prescription**	4	2	
**Drug formulations reported to be used:**			
Separate drugs	6	3	
2-drug Fixed Dose Combinations	0	2	
3-drug Fixed Dose Combinations	2	2	
4-drug Fixed Dose Combinations	32	40	1
No response	6	11	1
**Prescription of Streptomycin**	6	7	
**Adequate duration (6 months)**	19	18	1
**Shorter duration(less than 6 months)**	2	6	
**Longer duration (more than 6 months)**	25	25	1
**Duration not mentioned**	3	6	
**Use of Levofloxacin**	3	1	

In response to the question on a prescription for a patient known to be suffering from MDR-TB, there were even more discrepancies. These are summarized in Table 4. Only 5 of the 106 respondents could write an appropriate prescription with a minimum of 3 new second line- drugs in the right doses for a minimum recommended duration of 18 months. 13 returned prescriptions that continued first line drugs without adding any second line drugs, 25 returned blank prescriptions in response to this question and 35 of respondents added a single second line drug. In 70% of the prescriptions, this was a fluoroquinolone.

**Table 4:** Analysis of the physicians' prescriptions for MDR TB.*

Number of second line drugs in the prescription	Number of doctors prescribing	Number of prescriptions with appropriate regimens
5	4	1
4	5	1
3	8	3
2	16	3
1	35	0
0	13	0

*25 prescriptions were blank.

## Discussion

India accounts for about one-fifth of the global burden of TB[6]. The DOTS-based RNTCP has been remarkably successful in achieving the global targets of detecting 70% of the estimated TB cases and curing 85% of them[5], [6]. In spite of a significantly strengthened TB programme and the progress made, 50–80% of TB patients in India still seek care at private clinics and TB treatment offered in the private health sector remains substandard[11], [12], [13]. A study conducted at our hospital among TB patients, not exposed to TB services offered in the public sector, found 170 out of 200 patients interviewed (85%) to be unaware of the DOTS programme.[14].This is further evident from the fact that out of the total market of USD 94 million for the first-line anti-TB medicines in India, the public sector purchases drugs worth USD 24 million while the private sector accounts for the remaining USD 61 million[15].

The RNTCP has made several attempts to engage the private health sector in general and private practitioners in particular in TB care and control[2], [16]. Based on numerous successful projects with documented evidence on feasibility, effectiveness and cost-effectiveness[17], [18], the RNTCP has designed and promoted public-private mix schemes that define the input of the public sector and expectations from the private sector and offer financial and non-financial incentives[2]. Several thousands of private practitioners as well as non-governmental organizations are reported to have been collaborating under the various schemes[5].However, they comprise only a miniscule of the large private sector in the country and their precise contribution to detecting and treating TB cases is not known.

In the 1991 study of prescribing behaviour of private practitioners, 100 doctors reported to have provided 80 different prescriptions[1]. A similar study undertaken in Mumbai and rural Pune, a neighbouring district and published in 1998, reported 105 private practitioners giving 79 diverse prescriptions[11] and in this study, 106 doctors wrote 63 different prescriptions. Can this be called progress? An analysis of the prescriptions may provide some measure of consolation. Seventy three doctors used four-drug fixed dose combinations and 53 wrote prescriptions of drugs for longer than six months suggesting an overkill rather than under-treatment of their TB patients. If the patients did take their medicines as prescribed and did adhere to treatment, a large majority were less likely to develop multidrug-resistance. However, the above referred study that followed up patients diagnosed in the private sector showed that patients did not generally take all the medicines as prescribed by their doctors and that their treatment completion rates were very poor[11].

We extended the senior author's original study by also auditing the prescriptions of the same group of physicians for MDR-TB. There were several reasons for doing this. The prevalence of MDR-TB has clearly grown significantly in India over the last two decades, accounting for 20% of the global MDR-TB cases reported in 2006[19]. The actual levels of MDR-TB may be much higher than those projected by national estimates as the patients diagnosed and managed in the private sector never get reported. A recent study in Mumbai found prevalence rates of MDR TB to be 24% among newly diagnosed, previously untreated patients and rates of 41% among first-line drug failures[20].

Poor prescribing practice is a major factor fuelling the MDR-TB epidemic. A report from Mumbai showed that about 10% of all MDR-TB cases were XDR-TB[21]. The quality of prescriptions for MDR-TB was even more dismal than those for drug-susceptible TB. Only 5 of the 106 respondents could write an appropriate prescription with a minimum of 3 new second-line drugs in the right doses for a right duration.

Over a third of respondents added a single second-line drug only, this single drug being a fluoroquinolone in 70% of such prescriptions. It is not surprising that a recent study from our center reported 30% of all MTB cultures to be fluoroquinolone resistant[22]. This combination of MDR and additional fluoroquinolone resistance — pre-XDR TB — is a direct result of inappropriate and indiscriminate fluoroquinolone use. A majority of the prescriptions reported by the practitioners would serve only to amplify resistance.

An important limitation of this follow up study, especially with regards to its comparability with the original study, needs to be acknowledged. The original 1991 study was a one-on-one study conducted among a randomly selected cohort of 143 physicians chosen from a list of 287 physicians, while the present study used a CME session for the purpose. This may have introduced a “volunteer bias” with a likelihood of only those doctors who needed to update their knowledge or those who had the time available attending the CME. However, to our knowledge, the doctors attending CMEs offered regularly at our hospital, is always a mix of not only those seeking to gain new knowledge but also the “good and regular” ones wishing to keep their knowledge up-to-date. It includes doctors with large as well as small practices. Also, our conducting CMEs generally on a Sunday facilitates attendance of doctors who are otherwise busy with their practices on week days. Furthermore, conducting one-to-one interviews with practicing doctors is much more time and resource intensive than it perhaps was two decades ago. Finally, even if we restrict the comparability of the two studies to simply drawing a sample of doctors from the same geographical area with predominantly slum population and a very high prevalence of TB, the findings are still worrisome and demand attention of all those concerned with TB control in the city and the country. Another limitation of this study, as it was of the original study, is that what the doctors reported could only be said to reflect their knowledge and not necessarily their practices.

It may be unreasonable to expect the RNTCP to effectively reach hundreds of thousands of practicing doctors. At the same time, continued mismanagement of TB and MDR-TB patients by private practitioners despite efforts to engage them can potentially undo all the efforts and achievements of RNTCP. How can this worrisome situation be addressed? Training and orientation of practicing doctors by both RNTCP experts and private chest physicians who believe in evidence-based practice is of course essential but may not be sufficient. Some of the recommendations of a recent joint monitoring mission of the RNTCP are also worth reiterating. They include reviewing RNTCP's current approaches to engage the private sector in TB care and control and redesigning them through strategic consultations with key stakeholders such as allopathic and non-allopathic professionals' associations and non-governmental organizations, pharmaceutical industry, pharmacies and consumer organizations. Assessing ways to set-up a system of mandatory notification is also recommended.

Given the magnitude of the problem of TB and MDR-TB and the size of the private sector in India, ignoring the private sector or dealing with it in a superficial manner cannot be an option. Evidence from numerous successful small projects shows that local public-private initiatives do work well[17]. Willingness on the part of the RNTCP staff to initiate and foster collaboration is a key requirement. A local intermediary organization acceptable to both parties, if available, hastens the process. A logical first step that each public sector tuberculosis unit can take would be to identify willing practitioners and support development of a few best practice clinics and hospitals in the private sector for management of TB and MDR-TB. Using the private sector to help achieve universal access to rational and standardized TB care can be a win-win situation not only for programme managers and private providers but also for patients with TB and people at large. Furthermore, some thorny issues must be addressed as a priority. Currently, the RNTCP tends to collaborate only with those individual and institutional providers who agree to follow the programme recommendations. As a result, those who follow appropriate, rational, internationally recommended practices that do not exactly match the programme guidelines cannot receive any support or recognition from the programme. Specifically, as the programme uses intermittent treatment regimens, physicians using daily regimens correctly to treat their patients cannot hope to collaborate with the programme.

Considering their large numbers and diverse backgrounds, it would simply be not possible for the RNTCP to adequately orient, support and supervise hundreds of thousands of private practitioners. Parallel mechanisms should be developed within the private sector to support itself to manage TB patients properly and pass on all the relevant information essential for surveillance to the RNTCP. This will require a combination of measures including support to intermediary professional associations and non-governmental institutions, promoting collaborative schemes and introducing regulatory approaches to make standardized TB and MDR-TB care widely accessible but only through “RNTCP-approved” outlets such as the clinics of certified private practitioners and accredited private institutions. If replacing the TB epidemic by a MDR-TB epidemic is to be avoided, the RNTCP will have to invest and help extend its own successes to the private sector. Measureable progress will be possible only with the creation of structures and staff to work with the private sector at the national, state and district levels. Half-hearted approaches are unlikely to make any significant change in the plethora of treatment regimens proffered by private practitioners even after two decades hence.
